# Assessment of air dose distribution in the vertical plane for better occupational exposure management

**DOI:** 10.1093/rpd/ncae150

**Published:** 2024-11-14

**Authors:** Tomuhiro Noro, Minoru Osanai, Shota Hosokawa, Maiko Kitajima, Megumi Tsushima, Kohsei Kudo

**Affiliations:** Japanese Red Cross Ishinomaki Hospital, 71 Nishimichishita, Hebita, Ishinomaki, Miyagi, 986-8522, Japan; Hirosaki University Graduate School of Health Sciences, 66-1 Hon-cho, Hirosaki, Aomori 036-8564, Japan; Hirosaki University Graduate School of Health Sciences, 66-1 Hon-cho, Hirosaki, Aomori 036-8564, Japan; Hirosaki University Graduate School of Health Sciences, 66-1 Hon-cho, Hirosaki, Aomori 036-8564, Japan; Hirosaki University Graduate School of Health Sciences, 66-1 Hon-cho, Hirosaki, Aomori 036-8564, Japan; Hirosaki University Graduate School of Health Sciences, 66-1 Hon-cho, Hirosaki, Aomori 036-8564, Japan; Hirosaki University Graduate School of Health Sciences, 66-1 Hon-cho, Hirosaki, Aomori 036-8564, Japan

## Abstract

The International Commission on Radiological Protection recommended a significant reduction of the equivalent dose limit for the eye lens. Reportedly, medical staff in charge of diagnostic imaging procedures may exceed the new dose limits for the eye lens. The use of dosimeters dedicated to the eye lens remains low, and dosimeters for the neck region were often used to assess eye lens doses. However, measurements by neck badges may overestimate or underestimate the recommended eye lens doses because the height of the neck differs from that of the eye. This study aimed to evaluate the air dose distribution in the vertical plane to understand the difference between neck and eye doses. H^*^(10) in the height of the eye position was 52.8% lower than that in the height of the neck position in the under-table position. Thus, the equivalent eye lens dose evaluated using a neck badge dosimeter may be overestimated.

## Introduction

In 2011, the International Commission on Radiological Protection recommended a significant reduction in the equivalent dose limit for the eye lens [1, 2]. Reportedly, medical staff in charge of imaging procedures (e.g. interventional radiology and computed tomography) may exceed the new dose limits [3–5]. The importance of radiation protection has increased [6, 7], and the use of dosimeters dedicated to the eye lens remains low. Dosimeters for the neck region were often used to assess eye lens doses. However, measurements by neck badges may overestimate or underestimate eye lens doses because the height of the neck differs from that of the eye. Therefore, scattered X-rays considering the difference in height might be evaluated by developing an air dose distribution map for the perpendicular surface to the floor. It is also necessary to identify the source of the scattered X-rays for more appropriate dosimetry and provide appropriate radiation protection for each source. The source of the scattered X-rays might be visualized using a homemade lead pinhole camera (based on the principle of the pinhole camera) to identify the source of the scattered X-rays.

This study aimed to evaluate the air dose distribution in the vertical plane to determine the difference between neck and eye doses while identifying the source of scattered X-rays.

## Materials and methods

### Identification of the source of scattered X-rays

**Figure 1:**
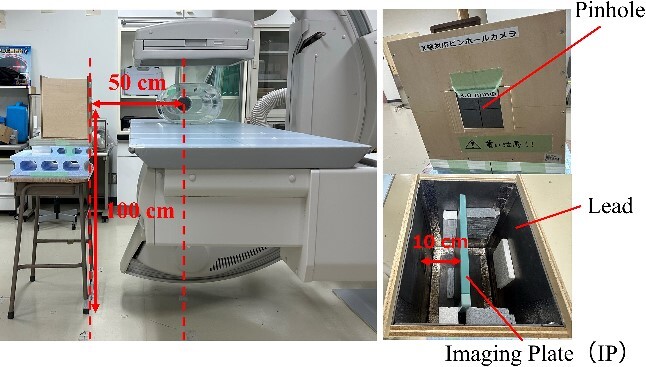
Layout of lead pinhole camera.

We identified the locations where scattered X-rays are mainly generated using a pinhole camera covered by a 2-mm-thick lead. The pinhole area is constructed with a lead thickness of 1 mm and a pinhole diameter of 3 mm. As shown in Fig. 1, a lead pinhole camera was set up so that the pinhole was 50 cm away from the center of the irradiation field at a height of 100 cm. The imaging plate (8 × 10 inch, 20.3 cm × 25.4 cm) was fixed 10 cm away from the pinhole in the lead pinhole camera. The patient phantom was irradiated by the X-ray generator (Ultimax-I DREX-UI80, Canon; maximum anode heat capacity is 600 kHU), which is the same type used in actual fluoroscopy or IR. The locations of scattered X-rays in the under-table and over-table positions were identified via pinhole images. The imaging conditions used to obtain the required pinhole image density were as follows: tube voltage: 80 kV, tube current: 100 mA, irradiation time: 1 s, and the number of irradiations: 15. Because fewer scattered X-rays pass through the pinhole, the higher S-value was set than in usual computed radiography. The height of the couch from the floor during the under-table position was 100 cm, and the SID (source imaging receptor distance) was 100 cm. The height of the couch from the floor during the over-table position was 85 cm, and the SID was 115 cm.

### Development of the air dose distribution map of the surface that is perpendicular to the floor

**Figure 2:**
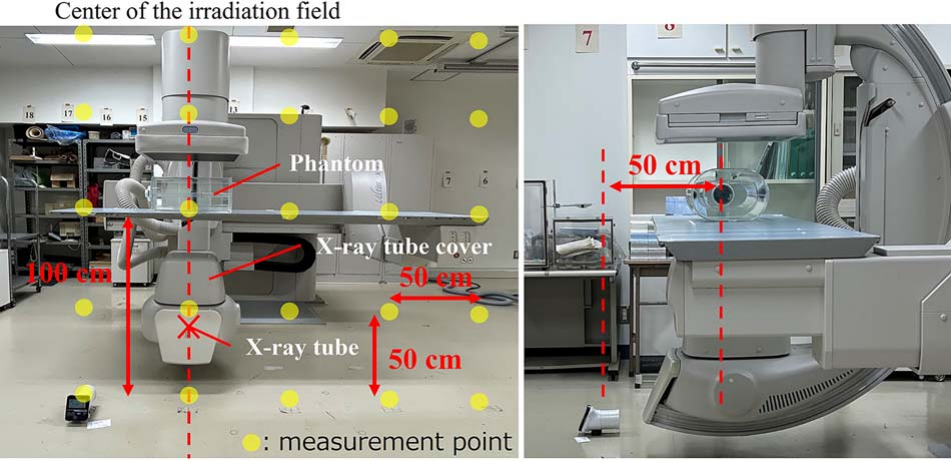
Layout of scattered X-ray measurement in the under-table position.

**Figure 3:**
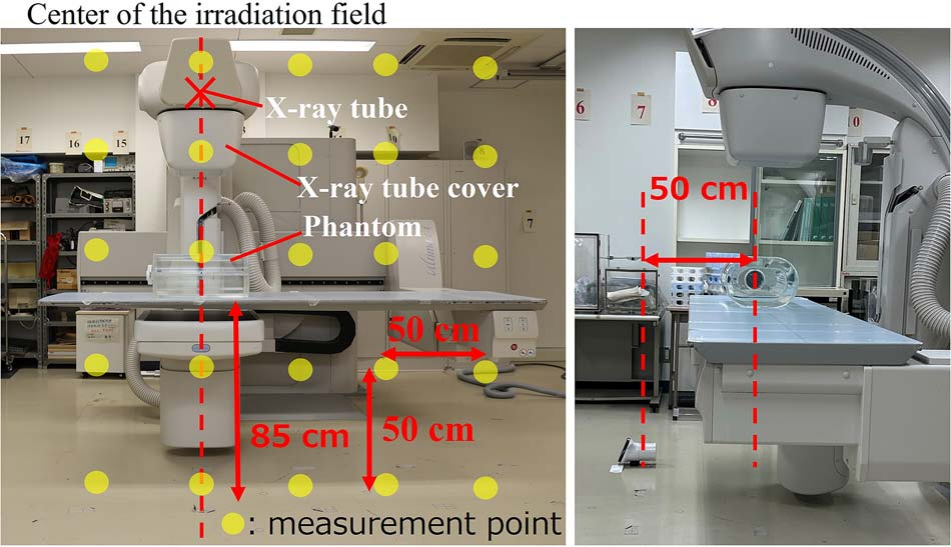
Layout of scattered X-ray measurement in the over-table position.

We have developed an air dose distribution map for the surface that is perpendicular to the floor to understand the difference between neck and eye doses. As shown in Figs 2 and 3, H^*^(10) for the area of 200 cm × 200 cm at the cross point of 50 cm × 50 cm, 50 cm away from the center of the irradiated field, was measured using an ionization chamber (ICS-1323, Hitachi) (effective measurement range: 0.3 μSv–10 Sv, 1.00 μSv/h–1.00 Sv/h) in the under-table and over-table positions. H^*^(10) every 25 cm at the line 50 cm away from the center of the irradiation field was also measured. The bottom line of the dosimetry surface was set at a height of 7 cm from the floor (the center of the ionization chamber is located 7 cm from the floor), and this line was assumed to be the floor surface during X-ray irradiation. H^*^(10) with a lead protective curtain (substituted with a lead apron: 0.5 mmPb) attached to the couch in the under-table position was also measured to evaluate the attenuation effect of scattered X-rays when a lead curtain was used. A dose distribution map was developed with an interpolation software program (Visualizer Pro, Malloc Code) from the measurement results. The exposure parameters were as follows: tube voltage: 80 kV, tube current: 25 mA, and irradiation time: 500 ms. The height of the couch from the floor was 100 cm, and the SID was 100 cm in the under-table position. The height of the couch from the floor was 115 cm, and the SID was 85 cm in the over-table position.

## Results and discussion

### Identification of the source of scattered X-rays

**Figure 4:**
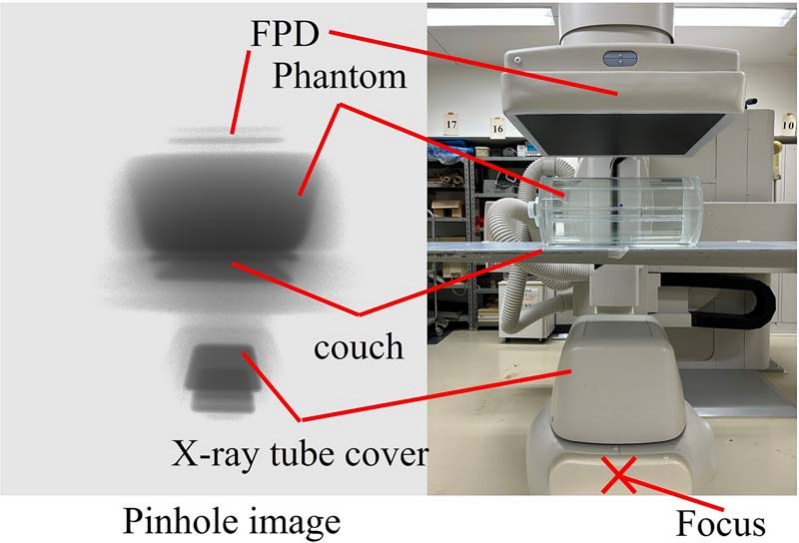
Identification of the source of scattered X-rays in the under-table position. (The position of the pinhole image differs from that of the optical photograph.)

**Figure 5:**
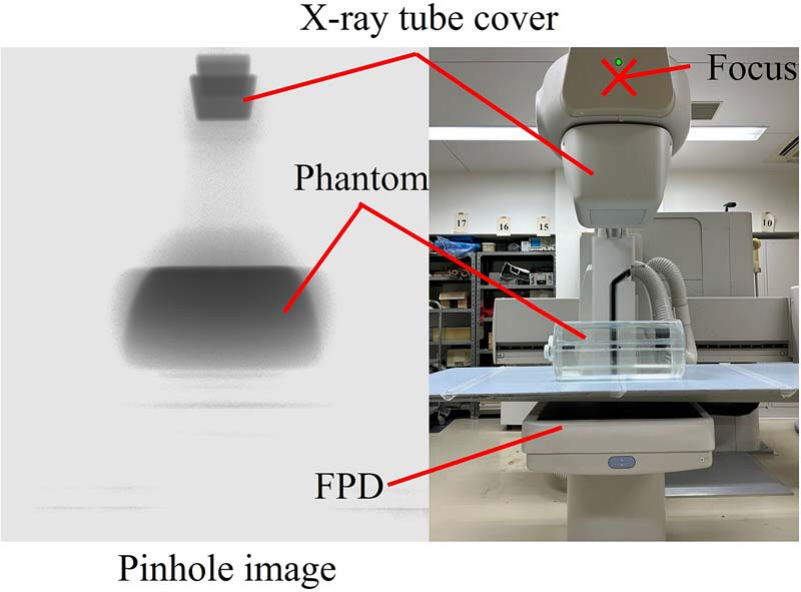
Identification of the source of scattered X-rays in the over-table position. (The position of the pinhole image differs from that of the optical photograph.)

Figures 4 and 5 show pinhole images taken in the under-table and over-table positions. Scattered X-rays were rendered black in the pinhole image, and the areas of high scattered X-ray doses were dense. The main source of scattered X-rays was the phantom in the under-table and over-table positions. Scattered X-rays from the couch and FPD (flat panel detector) were also observed in the under-table position. Generally, scattered X-rays from the patient were high; however, it was generated by the X-ray tube cover. Therefore, it is also necessary to take measures for scattered X-rays from the X-ray tube cover, couch, and FPD.

### Development of the air dose distribution map for the surface that is perpendicular to the floor

**Figure 6:**
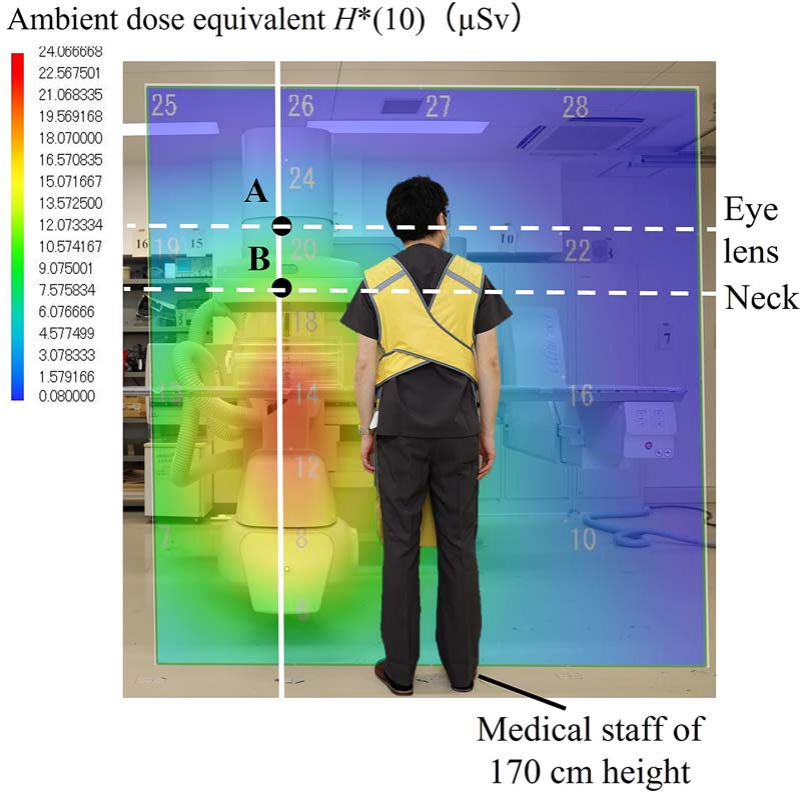
Comparison of H^*^(10) of the neck and eye in the under-table position.

**Figure 7:**
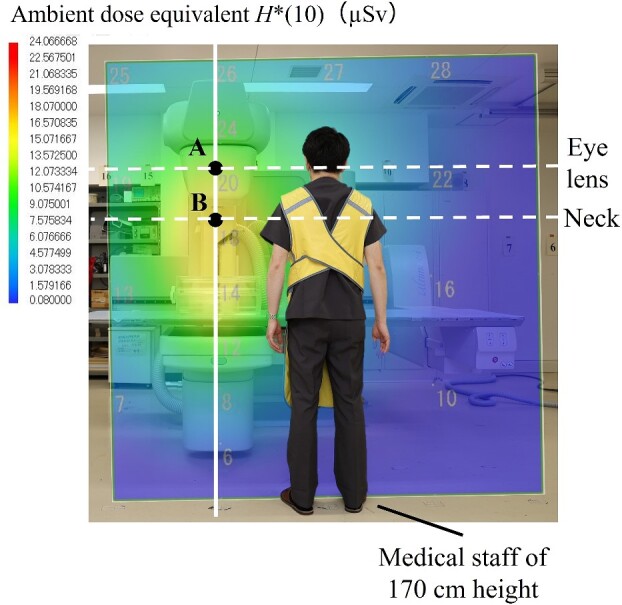
Comparison of H^*^(10) of the neck and eye in the over-table position.

**Figure 8:**
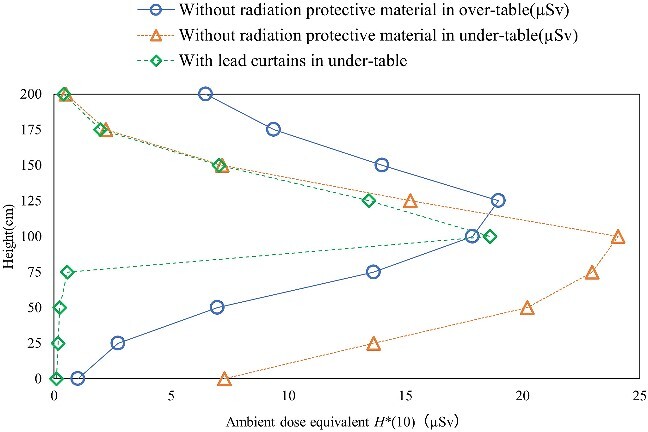
Measurement results of H^*^(10) at the center axis of the irradiation field, 50 cm away from the phantom.

Figures 6 and 7 show the air dose distribution map for the surface that is perpendicular to the floor in the under-table and over-table positions. The figures also show a medical staff member who is 170 cm tall. Figure 8 shows the measurement results of H^*^(10) at the line 50 cm away from the center of the irradiation field. The dose distributions of the under-table and over-table positions were high near the phantom, between the phantom and the X-ray tube. The maximum air dose in the under-table position condition was 24.1 μSv (air dose per irradiation for the irradiation conditions at this time) at a height of 100 cm at the center of the irradiation field. The maximum air dose during over-table position irradiation was 19.0 μSv at a height of 125 cm at the center of the irradiation field. In each of the settings of under- and over-table positions, the high dose levels are consistent with the results of the source of scattered X-rays observed by the pinhole camera. The maximum dose in the over-table position was lower than that in the under-table position because of the long distance between the X-ray tube and the phantom in the over-table position compared to that in the under-table position. H^*^(10) at the height of 150 cm (eye position) was 52.8% lower than that at the height of 125 cm (neck position) in the under-table position [as no significant difference is observed between the 3 mm dose equivalent and the 1 cm dose equivalent for the X-ray energies used in this study, a comparison is made using H^*^(10)]. H^*^(10) at a height of 150 cm was 26.2% lower in the over-table position than that at a height of 125 cm. Therefore, the equivalent dose of the eye lens evaluated using a neck badge dosimeter might be overestimated. Wearing protective eyewear will aggravate the overestimation. For more appropriate management, medical staff near high-dose areas should wear dedicated personal dosimeters for the eye lens.

**Figure 9:**
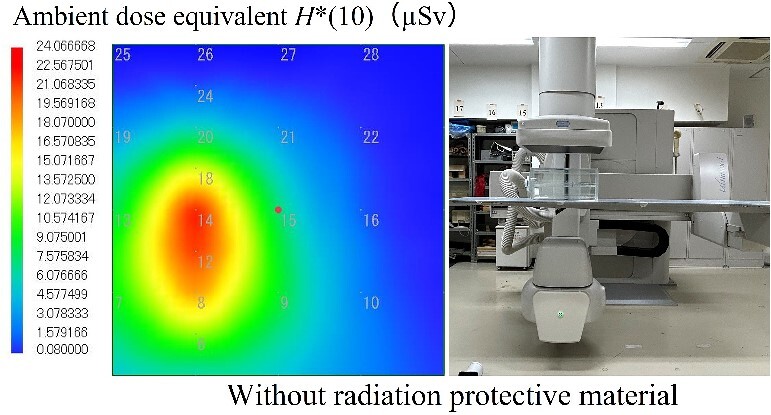
The air dose distribution map for the surface that is perpendicular to the floor without radiation protective material in the under-table position.

**Figure 10:**
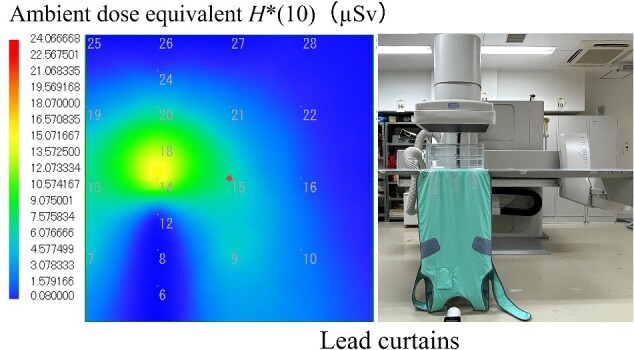
The air dose distribution map for the surface that is perpendicular to the floor with lead curtains in the under-table position.

Figures 9 and 10 show the air dose distribution maps for the surface that is perpendicular to the floor without radiation-protective material and with lead curtains in the under-table position. The lead curtains can decrease the high-dose area. The dose attenuation ratio of the lead curtain was 97.5%–98.8% below 75 cm high and 1.86%–22.7% above 100 cm high. Also, the amount of scattered X-rays not only from the phantom but also from the X-ray tube cover was decreased with the lead curtains. Therefore, lead curtains are useful as a radiation protection measure during diagnostic imaging procedures.

However, this study had some limitations. An incident dose to the imaging plate and pixel value in the images of computed radiography in the device used in this study are not proportional. Therefore, rigorous quantitative analysis of pinhole images is difficult, and evaluation of the relationship between the dose of scattered X-rays and pixel value is an issue for the future. Furthermore, herein, the neck and eye heights were set at 125 and 150 cm, respectively; however, the actual heights could be different, in which case the dose ratios would be slightly different. In positions far from the irradiation field, the difference in dose distribution due to height may be small, according to geometrical relationships. In this study, only two angles (i.e. the over-table and under-table positions) of verification were performed. It may be necessary to validate other angles, such as the left anterior oblique and right anterior oblique.

## Conclusion

In this study, a lead pinhole camera was used to identify the source of scattered X-rays. In addition, the air dose distribution map for the surface that is perpendicular to the floor was evaluated to understand the difference between neck and eye doses. The main source of scattered X-rays was the phantom; however, it is also necessary to take measures against scattered X-rays from the X-ray tube cover, couch, and FPD. In the air dose distribution map for the surface that is perpendicular to the floor, H^*^(10) in the height of the eye position was lower than that in the height of the neck position. Therefore, there is a possible overestimation of diagnostic imaging procedures close to the irradiated field, such as the surgeon when the eye lens dose is estimated by the neck badge. Also, lead curtains are useful as radiation protection during diagnostic imaging procedures because they can decrease the high-dose area, and the amount of scattered X-rays not only from the phantom but also from the X-ray tube cover was lower with the lead curtains.
